# Harnessing the Antioxidative Potential of Dental Pulp Stem Cell-Conditioned Medium in Photopolymerized GelMA Hydrogels

**DOI:** 10.34133/bmr.0084

**Published:** 2024-09-17

**Authors:** Shuntaro Yamada, Niyaz Al-Sharabi, Francesco Torelli, Ana Angelova Volponi, Linda Sandven, Minoru Ueda, Inge Fristad, Kamal Mustafa

**Affiliations:** ^1^ Center of Translational Oral Research, Department of Clinical Dentistry, Faculty of Medicine, University of Bergen, Bergen, Norway.; ^2^ Centre for Craniofacial and Regenerative Biology, Faculty of Dentistry, Oral & Craniofacial Sciences, King’s College London, London, UK.; ^3^ The Molecular Imaging Center, Department of Biomedicine, Faculty of Medicine, University of Bergen, Bergen, Norway.; ^4^ Department of Oral and Maxillofacial Surgery, Graduate School of Medicine, Nagoya University, Nagoya, Japan.; ^5^ Saiseiken Co. Ltd., Tokyo, Japan.

## Abstract

Gelatin methacryloyl (GelMA) stands out for its biocompatibility, tunability, and functionality, being often selected as a scaffolding material. However, the biological modulations induced by its photocrosslinking process on mesenchymal stem cells as well as stress mitigation measures remain insufficiently explored. By using GelMA of Good Manufacturing Practice (GMP) grade, this study aimed (a) to achieve a comprehensive understanding of the biological effects of photocrosslinking process with a specific focus on oxidative stress and (b) to develop a strategy to mitigate the adverse effects by employing conditioned medium (CM) by dental pulp stem cells (DPSCs). Following photocrosslinking, pathways related to oxidative phosphorylation and DNA repair were enriched in the presence of DPSC-CM carrying various antioxidants such as peroxiredoxin (PRDX) 1–6 and superoxide dismutase type 1 (SOD1), while the control samples exhibited enrichment in inflammatory signaling pathways. Incorporating DPSC-CM into the hydrogel notably reduced the degree of cellular oxidation caused by photocrosslinking and stress responses, resulting in improved cell viability, growth, motility, and osteogenic differentiation, as well as fewer apoptotic and senescent cells compared to those without DPSC-CM. The deteriorated biocompatibility of freshly crosslinked GelMA hydrogel was confirmed by the disrupted vasculature of chorioallantoic membranes in chicken embryos after implantation, which was prevented by DPSC-CM. In conclusion, this study demonstrates the robust antioxidative effects of DPSC-CM, mitigating the negative effect of GelMA photocrosslinking processes.

## Introduction

Gelatin methacryloyl (GelMA), a biomaterial derived from gelatin modified with methacrylate groups, has gained significant attention for tissue engineering applications for its cost-effectiveness, tunability, biocompatibility, and photocrosslinkable properties [1]. GelMA hydrogel can be photocrosslinked in the presence of a free radical-generating photoinitiator. This is enabled by the acrylate groups linked to the gelatin structure, which undergo radical chain polymerization. Among photoinitiators, lithium phenyl-2,4,6-trimethylbenzoylphosphinate (LAP) is known for its notable water solubility and effectiveness in facilitating rapid photopolymerization [2]. LAP exhibits its peak absorption within the ultraviolet A spectrum. However, its extended absorption into the visible spectrum allows for photoactivation through high-energy visible light sources like dental curing lamps, making it convenient for chair-side applications. Thereby, GelMA hydrogel has emerged as a promising scaffolding material for engineering various tissues, including dental, orofacial, and skeletal tissues, in future clinical settings [3].

Dental tissues have been indicated as a highly accessible source of mesenchymal stem cells (MSCs) for cell-based regenerative therapies. Dental stem cells can be obtained from tissues usually discarded during routine dental procedures, such as extraction of third molars, root canal treatment, periodontal surgery, or exfoliated deciduous teeth. Dental stem cells, originally derived from neural crest during embryonic development, are capable of differentiating into multiple lineages such as odontoclasts, osteoblasts, chondrocytes, adipocytes, myocytes, and neural cells in vitro [4]. Dental pulp stem cells (DPSCs) are among the most commonly used dental stem cells, demonstrating a higher proliferation rate and comparable or even superior osteogenic potentials compared to MSCs from well-established sources such as bone marrow or adipose tissues [5]. Furthermore, DPSCs secrete a range of bioactive substances, such as growth factors, cytokines, and chemokines, which regulate multiple biological signaling processes [6]. Owing to their regenerative characteristics, DPSCs have been used in combination with various biomaterials to generate biomimetic constructs for cell therapies.

DPSC-laden GelMA hydrogels have been tested for the regeneration of various tissues including dental pulp [7], bone [8,9], cartilage [10], and other oral and craniofacial tissues [11]. However, despite its known biocompatibility, a photocrosslinking process seems associated with cytotoxicity, although the degree varies depending on crosslinking conditions and cell type-specific sensitivity [2]. Upon photoinitiation, LAP breaks down and releases free radicals, which propagate the reaction of monomers and oligomers to be polymerized. Free radicals possess the ability to harm a wide range of biomolecules, such as DNA, proteins, and lipids. Elevated levels of reactive oxygen species (ROS) may therefore induce cellular damage and malfunction represented by cell death through apoptosis and necrosis, senescence, or even oncogenicity [12]. Under physiological conditions, ROS are primarily produced by mitochondrial complexes I and III, as well as the NADPH (reduced form of nicotinamide adenine dinucleotide phosphate) oxidase isoform NOX4, regulating various biological processes such as gene expression, enzymatic activity, survival, differentiation, and immunomodulation [12]. Upon exposure to excessive ROS, the expression of the antioxidant molecules are triggered by multiple signaling cascades, including nuclear factor erythroid 2–related factor 2 (Nrf2) pathway, p38 mitogen-activated protein kinase (MAPK) pathway, nuclear factor κ-light-chain-enhancer of activated B cell (NF-κB) pathway, phosphoinositide 3-kinase (PI3K)/Akt pathway, as well as autocrine/paracrine interleukins [12]. In detail, MSCs are known to produce antioxidative enzymes such as cytosolic superoxide dismutase (SOD1), mitochondrial superoxide dismutase (SOD2), catalase (CAT), and glutathione peroxidase 1 (GPX1) at higher levels to maintain lower intracellular ROS compared to more differentiated cells [13,14]. However, evidence suggests that undifferentiated MSCs have instead greater susceptibility to externally applied oxidative stress than their more differentiated counterpart, similar to their sensitivity to radiation [15]. This may impair long-term self-renewal and differentiation capacities of MSCs.

**Fig. 1.**
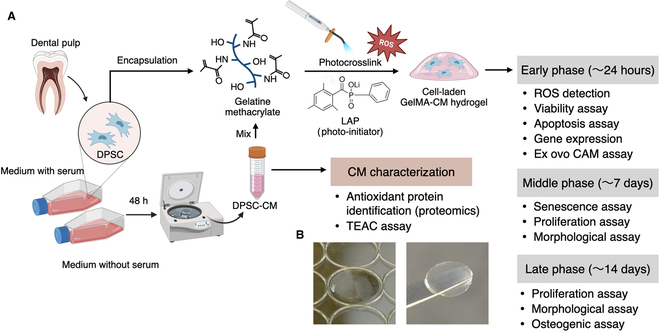
Schematic illustration of experimental design. (A) Secretome [i.e., conditioned medium (CM)] from dental pulp stem cells (DPSCs) was obtained and characterized by mass spectrometry-based proteomics. CM was then loaded together with DPSCs into a photocrosslinkable gelatin methacryloyl (GelMA) hydrogel to ease the oxidative stress. The oxidation and related biological events in DPSCs were then evaluated at different timepoints. (B) DPSC-laden GelMA hydrogels were prepared in a disc shape using a 96-well plate.

To date, there has been a lack of consideration and research investigating the potential resistance or biological reactions of DPSCs to oxidative stress applied during biofabrication, as well as the inherited antioxidative properties of these cells. In addition, despite the widespread use of photocrosslinkable hydrogels, the cellular responses to photocrosslinking processes have been underdocumented. In our prior work, we identified various antioxidant molecules secreted by bone marrow-derived MSCs (BMSCs) during in vitro cell culture and demonstrated that conditioned medium (CM) enriched with these antioxidants could mitigate cell dysfunction induced by hydrogen peroxide-induced oxidative stress [16]. The present study hypothesized that DPSCs, during cell culture, would also secrete abundant antioxidant molecules capable of alleviating oxidative stress induced by the photoinitiator during hydrogel polymerization. Our investigation involved an initial assessment of antioxidant molecules in DPSC-CM by mass spectrometry-based proteomics and comprehensive cellular and analyses of kinetics and functionality by using Good Manufacturing Practice (GMP)-grade GelMA hydrogels (Fig. 1A and B). The present study demonstrated the dynamic modulation of cell kinetics induced by photocrosslinking, the consequences of which persistently altered their functionalities and regenerative capacity. Notably, the functionalization of the hydrogels with DPSC-CM rescued the post-crosslinking negative effects, offering insights into the potential use of DPSC-CM in emerging tissue engineering applications.

## Materials and Methods

### DPSC isolation and expansion

The study was carried out in compliance with ethical standards, including obtaining written informed consent from the donors and receiving ethics approval from the Regional Committee for Medical and Health Research Ethics in Norway (REK project: 2009/610/REK vest).

Dental pulps were obtained from the wisdom teeth of 3 donors (aged between 18 and 24 years old) and were enzymatically digested by collagenase type 1 (4 mg/ml) and dispase (2 mg/ml) for 1 h at 37 °C prior to being plated into cell culture flasks. MSC characterization was performed by evaluating their multi-lineage differentiation potential and the expression profile of putative MSC markers. The isolated cells exclusively expressed MSC-positive markers, namely, CD73, CD90, and CD105, while lacking expression of CD34, CD45, and HLA-DR (Fig. S1). DPSCs were maintained in growth medium consisting of Dulbecco’s modified Eagle’s medium (DMEM; 10566016, Gibco, USA) supplemented with 10% fetal bovine serum (FBS; 10270-106; Gibco, USA) and 1% penicillin/streptomycin (SV30010; HyClone, USA) at 37 °C and 5% CO_2_ in a humidified condition. DPSCs at passages 3 to 5 were used in the study.

### Preparation of DPSC-CM

To generate DPSC-CM, the cells from 3 donors at passage 3 were seeded at a seeding density of 5 × 10^3^ cells/cm^2^ and cultured in the growth medium. When reaching 75 to 85% confluency, the cells were washed in phosphate-buffered saline (PBS) for 3 times and then cultured in serum-free DMEM for 48 h at 37 °C and 5% CO_2_ in a humidified condition. This corresponds to approximately 20 ml of DMEM conditioned by 8 to 10 million DPSCs. Upon medium collection, a centrifugation step was performed at 300 rcf for 5 min to obtain the supernatant, which underwent an additional centrifugation at 2,000 rcf for 20 min to remove residual cellular debris and apoptotic bodies. Subsequently, the supernatant was filtered using a filter with a pore size of 0.2 μm and stored at −80 °C. CM from 3 donors was pooled for the experiments except for a proteomics analysis.

### Proteomics and identification of antioxidant molecules in DPSC-CM

To determine the presence of antioxidant proteins in DPSC-CM, 10 ml of CM was concentrated to ≤300 μl by repeated centrifugation at 3,600 rcf using Amicon Ultra-15 Centrifugal Filter Units with Ultracel-100 membrane (molecular weight cutoff = 100 kDa; Merck Millipore, USA). A total of 20 μg was analyzed using liquid chromatography with tandem mass spectrometry. Subsequently, mass spectrometry (MS)-based proteomics data were searched in MaxQuant (version 1.6.1.0; Max Planck Institute for Biochemistry, Martinsread, Germany) against the concatenated forward and reverse decoy Swiss-Prot Homo sapiens database version. Then, Perseus version 2.0.3.1 software (Max Planck Institute of Biochemistry) was used to filter and process the protein expression in DPSC-CM. For identification of antioxidant proteins, the Antioxidant Protein Database (AOD), generated in accordance with UniProtKB/Swiss-Prot databases, was used by prescribing FASTA format sequences of the protein IDs in the AodPred search [17].

The amount of total protein in the naïve (i.e., unconcentrated) CM was measured by spectrophotometer at the absorbance of 280 nm. To evaluate the antioxidant activity of CM, the Trolox equivalent antioxidant capacity (TEAC; MAK187, Sigma-Aldrich, USA) assay was performed according to the manufacturer’s protocol. Briefly, CM was diluted 1:10 in Milli-Q water, and 100 μl of the sample solution and Trolox standard solution was aliquoted into each well of a 96-well plate. A total of 100 μl of Cu^2+^ working solution was added in the samples, which were then incubated for 90 min at room temperature. Absorbance was measured at 570 nm using a Varioskan LUX multimode microplate reader (VLBL00D0, Thermo Fisher Scientific, Finland). The concentration of Trolox equivalents was calculated as follows:

$$\frac{s_{a}}{S_{v}}=C$$

where *S_a_* is the amount of antioxidant (nmol) from the standard curve, *S_v_* is the sample volume (μl), and *C* is the concentration of antioxidant in the samples (nmol/μl).

### GelMA hydrogel reconstitution and 3D cell encapsulation

GelMA solutions were reconstituted by dissolving purified GelMA prepolymer powder (X-Pure GelMA 160P60 RG, Rousselot Biomedical, Belgium) in 100% (w/v) DMEM (21041025, Thermo Fisher Scientific, USA) or 50 to 100% (w/v) DPSC-derived CM in the presence of 2.4% (w/v) LAP (L0290, Tokyo Chemical Industries Co., Japan) on a magnetic stirrer at 37 °C under sterile conditions in a light-protected environment. The cell suspension was then added to the prepared hydrogel precursor solution in DMEM (CM0) or in CM in a ratio of 1:4 to obtain 2 experimental groups (40% CM: CM40; 80% CM: CM80). Totally, 50 μl of the premixed cell–GelMA solution was then transferred into each upper circular rim of the 96-well plate dish lid.

Three-dimensional (3D) cell encapsulation was performed at room temperature by using a dental curing lamp set for one single cycle of 20 s at a light intensity of 1,200 mW/cm^2^ positioned at a distance of 2 cm from the top surface of the cell-laden constructs. After photopolymerization, samples were transferred to 48-well plates and incubated at 37 °C and 5% CO_2_ for the growth medium or osteogenic medium [DMEM supplemented with 1% penicillin and streptomycin, 10% FBS, 10 nM dexamethasone (D4902, Sigma, USA), 10 mM β-glycerophosphate (G9422, Sigma, USA), and 173 μM l-ascorbic acid (A8960, Sigma, USA)]. Culture medium was refreshed every 48 h.

### Bulk RNA sequencing

One hour after photocrosslinking of the cell-laden GelMA hydrogel, the samples were immediately snap-frozen in liquid nitrogen and stored at −80 °C. Total RNA was extracted using a Maxwell 16 Cell LEV Total RNA Purification Kit (AS1280, Promega, USA) in accordance with the manufacturer’s protocol. For bulk RNA sequencing (RNAseq), conducted by Novogene Ltd. (Cambridge, UK), human mRNA sequencing libraries were generated from total RNA through a poly(A) enrichment method. The sequencing process was executed utilizing the Illumina Sequencing PE150 platform, generating 6G raw data for each sample equivalent to 20 million paired-end reads.

### Cell oxidation assay

To assess cellular oxidation, CellROX Green Reagent (C10444, Thermo Fisher Scientific, USA) was used. The reagent was added at 5 μM either in cell suspension before photocrosslinking or in the culture medium 24 h after photocrosslinking. The samples were incubated for 30 min at 37 °C and then washed 3 times with PBS followed by fixation in 4% paraformaldehyde (PFA) for 15 min at room temperature before detection.

The detection of oxidated cells was conducted by a flow cytometer and confocal microscope. For flow cytometry analysis, the GelMA hydrogel was digested by type 1 collagenase (1.5 mg/ml; 4197, Worthington Biochemical, USA) for 1 h at 37 °C to collect cells in pellet, and then the cells were resuspended in PBS. The fluorescence intensity was measured by Accuri C6 (BD BioSciences, USA) using a 488-nm laser detected by a 530/30-nm filter (FL1). For microscopic observation, the nuclei were counterstained by 4′,6-diamidino-2-phenylindole (DAPI; 1:2,500, D9542, Sigma-Aldrich, USA). The z-stack images of GelMA hydrogel were captured in depth by Leica SP8 confocal microscopy (Leica Microsystems, Germany) equipped with a 20× objective and displayed as maximum projection images generated by Fiji/ImageJ.

### Cell acidity and viability assays

Intracellular acidity and cell viability were evaluated by flow cytometry in suspension to preliminarily estimate the effect of LAP-induced oxidative stress on the cells. For intracellular acidity assay, DPSCs were cultured in the growth medium in the presence of a pH probe, fluorescein isothiocyanate (FITC)–tetramethyl rhodamine isothiocyanate (TRITC)–dextran 500 (10 μg/ml; FTD500, TdB Labs AB, Sweden), overnight. The cells were then trypsinized and resuspended in the growth medium with 2.4% LAP. The activation of LAP was performed as described in the section of cell encapsulation in GelMA hydrogels. The fluorescence was measured by the Accuri C6 flow cytometer using 488-nm laser before adding LAP, after adding LAP, and immediately, 1 h, and 3 h after photoactivation. For the viability assay, DPSC was subjected to LAP photoactivation as mentioned above, followed by incubation for 10 min with propidium iodide (PI) (1:500; P3566, Thermo Fisher Scientific, USA) at 4 °C before measurement on Accuri C6. DPSCs exposed to the light in the absence of LAP served as a negative control.

To validate the cell viability assay in the presence of GelMA, Live/Dead staining was performed. Immediately after photocrosslinking of the GelMA hydrogel, the samples were stained by using LIVE/DEAD Cell Imaging Kit (R37601, Thermo Fisher Scientific, USA) and incubated for 30 min at room temperature before image acquisition by an inverted epifluorescent microscope (Eclipse Ti, Nikon, Japan).

### Cell apoptosis assay

Cells undergoing apoptosis were detected after 24 h of photocrosslinking by terminal deoxynucleotidyl transferase–mediated deoxyuridine triphosphate nick end labeling (TUNEL). Briefly, the cells were fixed in 4% PFA for 15 min at room temperature and then permeabilized by 0.25% Triton X-100 in PBS for another 15 min at room temperature. TUNEL assay was performed using Click-iT Plus TUNEL Assay Kits for In Situ Apoptosis Detection (C10617, Thermo Fisher Scientific, USA) according to the manufacturer’s protocol. The nuclei were counterstained by DAPI at 1:2,500. The samples were imaged by the confocal microscope as z-stack images covering a depth of 500 μm.

### Cell senescence assay

Cellular senescence was assessed after 7 d of culture by the detection of SA-β-galactosidase, using a confocal microscope and flow cytometry. The samples were fixed in 4% PFA for 15 min at room temperature. For imaging, SA-β-galactosidase was detected by incubation in CellEvent Senescence Green (1:500; C10840, Thermo Fisher Scientific, USA) for 1 h at 37 °C followed by nuclear counterstaining with DAPI at 1:2,500. For flow cytometry analysis, the hydrogel was digested by the collagenase solution for 1 h at 37 °C after fixation in 4% PFA. The cells were then resuspended in 1% bovine serum albumin (BSA) in PBS and incubated in CellEvent Senescence Green (1:500; C10840, Thermo Fisher Scientific, USA) for 1 h at 37 °C. After the incubation, the cells were washed in 1% BSA in PBS, and the fluorescence was read by Accuri C6 using a 488-nm laser beam and a 585/40-nm filter (FL2).

### Cell proliferation assay

To assess cell proliferation, 5-ethynyl-2′-deoxyuridine (EdU) was used to detect cells synthetizing DNA for cell division. Samples were incubated with 30 μM EdU in the growth medium at 37 °C and 5% CO_2_ for 24 h in a humidified environment before sample collection on days 1, 3, and 7. The samples were then fixed in ice-cold methanol for 5 min at −20 °C. The detection of incorporated EdU to the nuclei was performed using Click-iT EdU Cell Proliferation Kit (C10337, Thermo Fisher Scientific, USA) according to the manufacturer’s protocol. The nuclei were counterstained by DAPI at 1:2,500. The samples were imaged by the confocal microscope as z-stack images covering a depth of 500 μm.

### Cytoskeletal staining and image analysis

Samples collected on days 7 and 14 were fixed in 4% PFA for 15 min at room temperature. After washing in PBS, filamentous actin and nuclei were stained by Alexa Fluor 488 phalloidin (1:250; A12379, Thermo Fisher Scientific, USA) and DAPI (1:2,500), respectively, for 1 h at room temperature. The images were acquired by the confocal microscope as a z-stack covering a depth of 500 μm. The orientation of the cell nuclei to the surface of the hydrogel was analyzed using the “Analyze Particles” function in Fiji/ImageJ.

### Alkaline phosphatase staining

Samples for alkaline phosphatase (ALP) staining were collected on day 7 of osteogenic induction and fixed in 4% PFA for 3 min. The samples were then incubated in a staining solution consisting of bromochloroindolyl phosphate–nitro blue tetrazolium substrate (B5655, Sigma-Aldrich, USA) and deionized water for 30 min at room temperature. To visualize the internal staining patterns, the stained samples were subsequently incubated in a 15% sucrose solution in PBS for 3 h, followed by incubation in a 30% sucrose solution in PBS overnight. Finally, the samples were cryosectioned into 80-μm-thick slices at −20 °C.

### Alizarin red S staining

Samples for Alizarin Red S staining were collected on day 14 of osteogenic induction and fixed in 4% PFA for 40 min, followed by thorough overnight washing in Milli-Q water. Mineral deposition was stained with 0.1% Alizarin Red S (A5533, Sigma-Aldrich, USA) for 20 min, followed by washing 6 times with Milli-Q water. For quantification, the stained samples were dried, and the dye was extracted in 200 μl of 100 mM cetylpyridium chloride overnight at room temperature. Absorbance was measured at 540 nm using the microplate reader.

### RT-qPCR array

For the reverse transcription quantitative real-time polymerase chain reaction (RT-qPCR) array, samples were collected 2 h after photocrosslinking to evaluate genes involved in antioxidant stress, and after 7 days of osteogenic induction to evaluate genes involved in the differentiation. The RNA extraction was performed in the same method as method for the bulk RNAseq. Reverse transcription was performed using a High-Capacity cDNA reverse Transcription Kit (4368813, Applied Biosystems, USA). RT-qPCR was performed using the StepOne real-time PCR system (4376357, Applied Biosystems, USA) with TaqMan Universal Master Mix (4352042, Applied Biosystems, USA). The amplification was carried out in the StepOnePlus Real-Time PCR System (Applied Biosystems, USA) with initial activation of the polymerase at 95 °C for 20 s, followed by 40 cycles of PCR at 95 °C for 1 s (denature) and 60 °C for 20 s (anneal and extend). The evaluated panels were tailored with references to TaqMan Array Human Oxidative Stress and Antioxidant Defense (4413255, Applied Biosystems, USA) and Human Osteogenesis (4418741, Applied Biosystems, USA).

### Chorioallantoic membrane assays and quantification

Fertilized eggs of *Gallus gallus domesticus* were obtained fresh from a local farmer and incubated at 37.5 °C for 3 d in a humidified condition using a rotating egg incubator. The egg contents, including chicken embryos, were then transferred into sterile weighing boats with 2 ml of PBS and further incubated at 37.5 °C for an additional 4 d. On embryonic day 7, 3 cell-laden GelMA hydrogels, prepared with and without DPSC-CM, were placed onto each chorioallantoic membrane (CAM). On embryonic day 9, the CAM was dissected and fixed in 4% PFA overnight for blood vessel quantification and subsequent histological analysis.

For quantification, the microscopic images were obtained using a stereomicroscope (M205C/MC170HD, Leica, Germany) and quantified using a deep learning-based image analysis software, IKOSA CAM Assay Application (KML Vision GmbH, Austria).

The samples were dehydrated through a series of graded ethanol solutions (70%, 80%, 95%, and 100%) and cleared in xylene. Subsequently, tissues were infiltrated and embedded in paraffin wax using a tissue processor. Paraffin-embedded tissue blocks were sectioned at a thickness of 5 μm using a microtome followed by dewaxing and rehydration through a series of graded ethanol solutions. The sections were stained with Harris hematoxylin for 5 min, followed by a rinse in tap water. Acid alcohol was used for differentiation. Eosin staining for 2 min was followed by dehydration in graded ethanol and clearance in xylene before mounting.

### Bioinformatics and statistics

For the data analysis of the RNAseq experiment, the generated .fastq files were processed using an open-source platform, Galaxy (EU-server). Prior to data analysis, sample quality was verified by FastQC. HISAT2 and featureCounts were used to align RNAseq data to a reference genome, GRCh38/hg38, and to generates count matrices, respectively. For statistical analysis and data visualization, SRPlot, R with DESeq2, EnhancedVolcano, and pheatmap packages were used. For gene set enrichment analysis (GSEA), the software GSEA 4.3.2 with h.all.v2023.2.Hs.symbols.gmt gene set database was employed.

For the data from RT-qPCR array, lists of identified proteins by proteomics and differentially expressed genes (DEGs) by RT-qPCR arrays were further analyzed using the R package gprofiler2 and the STRING database version 11.5. Functional enrichment was evaluated using the open-source software Cytoscape version 3.9.1 by referencing the Gene Ontology (GO) knowledgebase.

All data are represented as mean ± SEM unless otherwise specified. Statistical analyses not mentioned above were performed by Prism 9 (Dotmatics, USA). For multiple comparisons, the data were evaluated by one-way analysis of variance (ANOVA) followed by Tukey’s multiple comparisons test. A *P* value of <0.05 was considered statistically significant.

## Results

### Antioxidant molecules secreted by DPSCs during in vitro culture

**Fig. 2.**
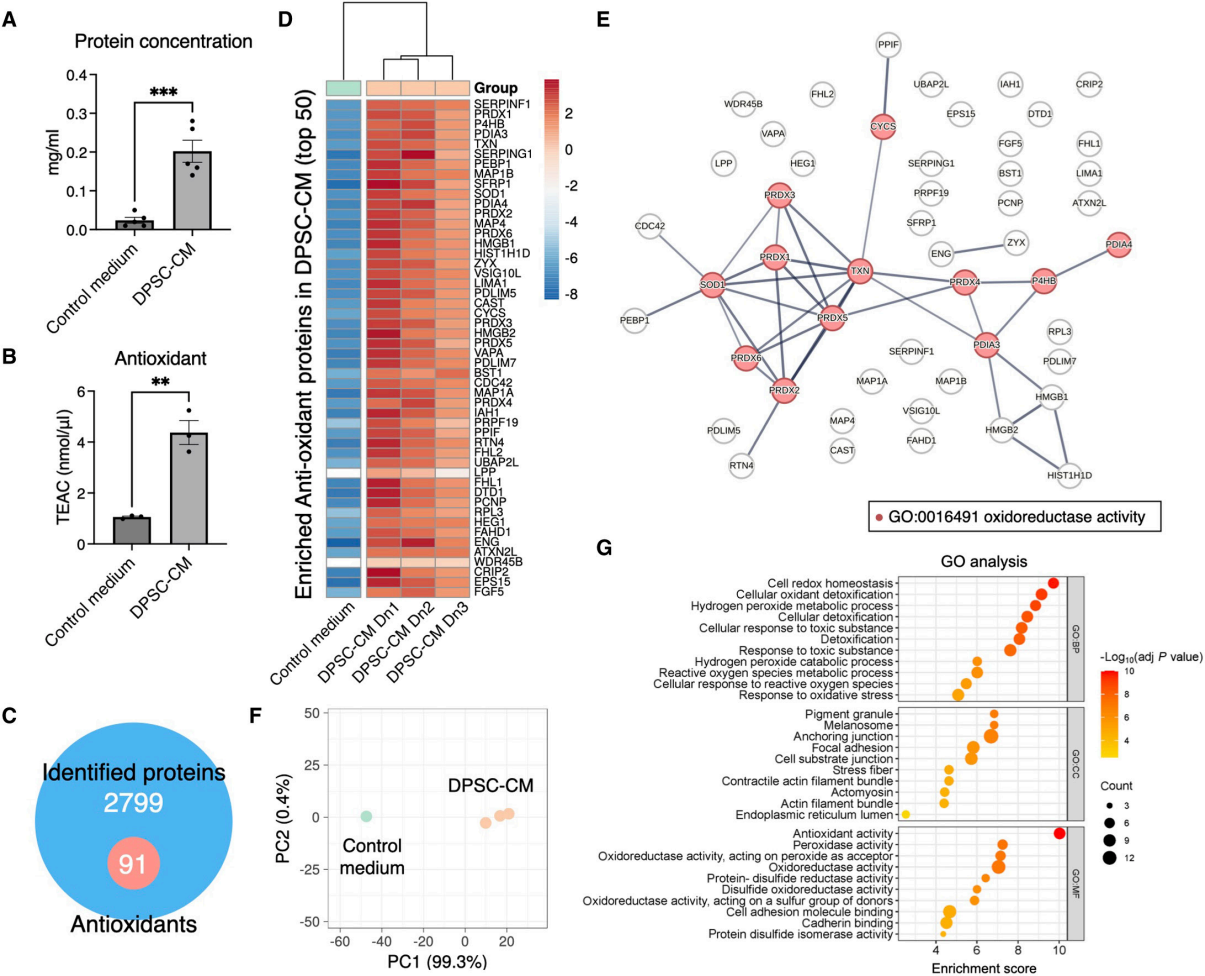
Antioxidative effects of DPSC-CM. (A) The concentration of protein in the control growth medium (i.e., DMEM) and DPSC-CM, showing an amount of total secreted proteins in DPSC-CM, while the control medium was mostly protein free. (B) The antioxidant effect of DPSC-CM was measured as Trolox equivalent antioxidant capacity (TEAC). (C) A mass spectrometry-based proteomics detected 2,799 proteins in DPSC-CM, 91 out of which were identified as antioxidants. (D) Heatmap of top 50 enriched antioxidant proteins in DPSC-CM. CM was produced using DPSCs from 3 different donors, showing a similar expression patterns. (E) Functional protein association network of the identified antioxidant proteins in DPSC-CM. In particular, oxidoreductase activity was predicted to be centered in the antioxidative effects of DPSC-CM. (F) Principal components analysis (PCA) of DPSC-CM from 3 donors, showing a similar trend in antioxidant protein expression among the tested subjects. (G) Gene Ontology (GO) analysis showing enriched biological process (BP), cellular component (CC), and molecular function (MF). **P < 0.01, ***P < 0.001 (Student’s *t* test).

In the current protocol for producing CM, DPSCs secreted a significant number and quantity of proteins into the culture medium, whereas the control medium was theoretically protein-free (Fig. 2A). The mean protein yield reached approximately 0.2 mg/ml in DPSC-CM (*P* = 0.0003). The TEAC assay confirmed that DPSC-CM exhibited an antioxidant effect equivalent to 4.4 nmol/μl of Trolox, which was significantly higher than the control medium (*P* = 0.0022; Fig. 2B).

The MS-based proteomics analysis identified 2,799 proteins in DPSC-CM (Fig. 2C). Among these, 91 proteins were identified to be antioxidant proteins by the AOD (Table S1) [17]. These included peroxiredoxin (PRDX) 1–6, SOD1, thioredoxin (TXN), and protein disulfide-isomerase A (PDIA) 3–4, which act as enzymatic antioxidants (i.e., oxidoreductases) (Fig. 2D and E). Despite some donor variance in the degree of secretion, the top 50 enriched antioxidant proteins were ubiquitously secreted by DPSCs isolated from all tested donors, and the secretion pattern was similar among the donors (Fig. 2F). GO enrichment analysis predicted that DPSC-CM would possess enriched antioxidant activity through the regulation of cell redox homeostasis, hydrogen peroxide metabolic processes via the activity of PRDX, TXN peroxidase, and peptide disulfide oxidoreductase (Fig. 2G).

### Significant alternation of gene expression profile in DPSCs after photocrosslinking

**Fig. 3.**
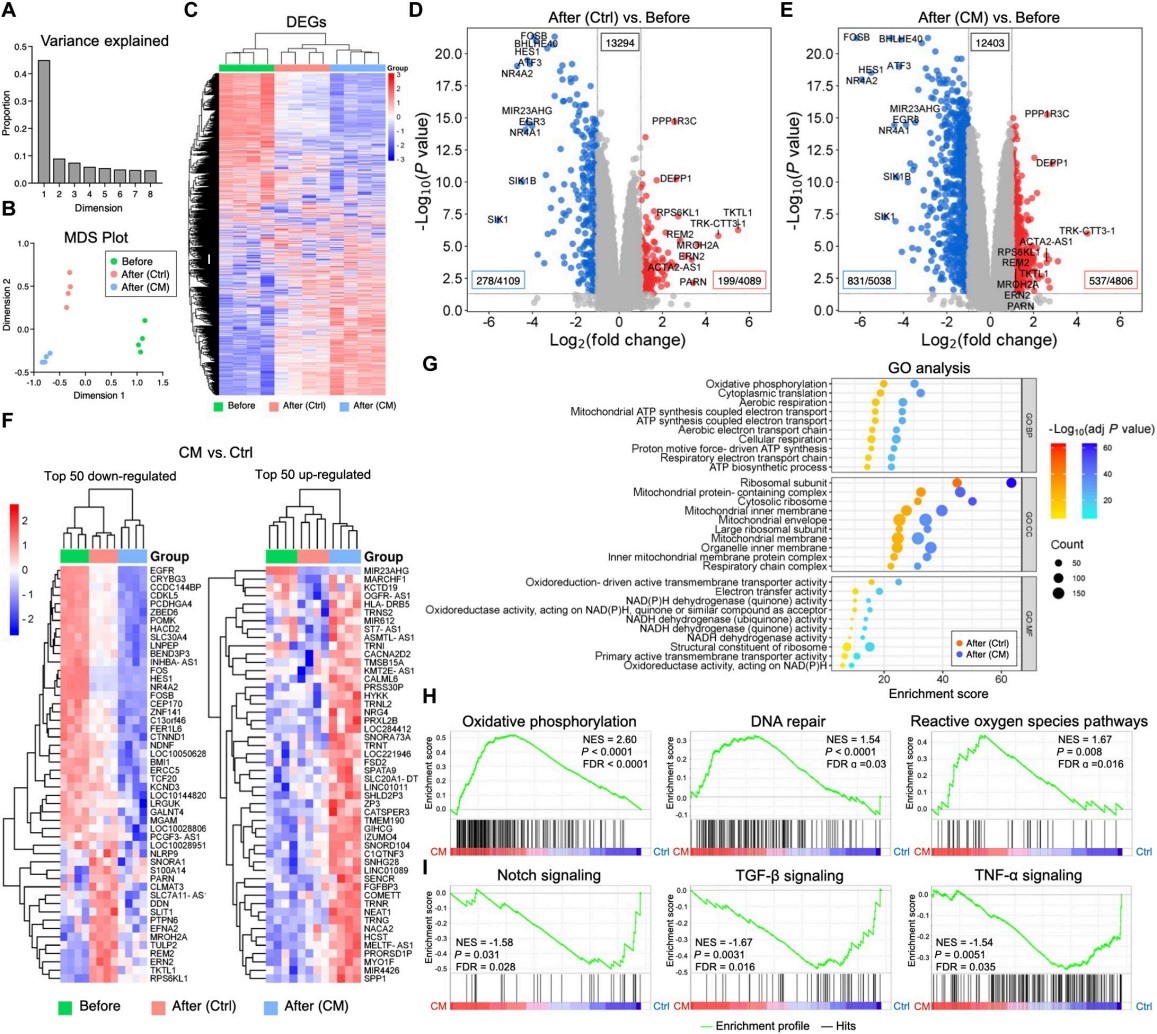
Bulk RNA sequencing of DPSCs before and after exposure to photoactivation (i.e., photocrosslinking) in GelMA hydrogel in the control medium (Ctrl) and DPSC-CM. (A and B) Multi-dimensional scaling (MDS) plot of replicated RNAseq samples. (C) Heatmap image showing differentially expressed gene (*P <* 0.05). (D) Volcano plots showing the differential expression analysis of the DPSC in freshly prepared GelMA consisting of the control medium and (E) that consisting of DPSC-CM versus the cells before photocrosslinking. (F) Heatmap images showing top 50 up-regulated and down-regulated genes in DPSCs in photocrosslinked GelMA with DPSC-CM compared to the control medium. (G) GO analysis showing the top 10 annotation of biological process (BP), cellular component (CC), and molecular function (MF) in DPSCs after photocrosslinking. (H and I) Gene set enrichment analysis (GSEA) of highly enriched representative pathways in DPSCs in GelMA with DPSC-CM (i.e., oxidative phosphorylation, DNA repair, and reactive oxygen species pathways) and DPSCs in GelMA with the control medium (i.e., Notch signaling, TGF-β signaling, and TNF-α signaling) using the Hallmark gene set.

To explore the gene expression profile of encapsulated DPSCs before and after photocrosslinking of GelMA hydrogels, bulk RNAseq was performed. Following photocrosslinking, significant alternation in gene expression profile was observed. Notably, the GelMA functionalization with DPSC-CM exhibited a pronounced impact on the expression profile, revealing a more pronounced pattern of enhanced up-regulation and down-regulation at a glance (Fig. 3A to C). Looking at DEGs, however, the majority of highly up-regulated genes after photocrosslinking in the control group (i.e., without DPSC-CM) were in fact down-regulated with the presence of DPSC-CM (Fig. 3D and E). Most down-regulated genes in the DPSC-CM group included signal receptors [e.g., epidermal growth factor receptor (EGFR)] and transcription factors (e.g., FOS and FOSB) in EGF/EGFR signaling and its regulatory signaling molecules (e.g., PTPN1, EFNA2, CTNND1, HES1, and BMI1) (Fig. 3F). Highly up-regulated genes in the DPSC-CM group were characterized by various mitochondrially encoded transfer RNA (tRNA; TRNS2, TRNI, TRNL2, TRNT, TRNR, and TRNG), tRNA metabolism regulator (PRORSD1P), and small nucleolar RNA (snoRNA: SNORA73A and SNORD104) as well as regulators of calcium ion mobilization (e.g., CACNA2D2, CATSPER3, and HCST). GO analysis revealed notable enrichments in oxidative phosphorylation, electron transport, and adenosine triphosphate (ATP) synthesis subsequent to photocrosslinking (Fig. 3G). The most up-regulated genes were linked to both ribosomal and mitochondrial units, indicating a pronounced involvement in activated oxidoreduction reactions and metabolism. Particularly, oxidative phosphorylation (*P <* 0.0001), DNA repair (*P <* 0.0001), and ROS pathway (*P* = 0.008) were enriched in the DPSC-CM group, shown by GSEA (Fig. 3H). Conversely, the control group exhibited enrichment in a known modulator of cell death associated with oxidative stress, namely, Notch signaling (*P* = 0.031), and inflammatory signaling pathways, including transforming growth factor-β (TGF-β) signaling (*P* = 0.0031) and tumor necrosis factor-α (TNF-α) signaling (*P* = 0.0051), were found enriched in the control group (Fig. 3I).

### Easing photocrosslinking-induced oxidative stress by DPSC-CM in the GelMA hydrogel

**Fig. 4.**
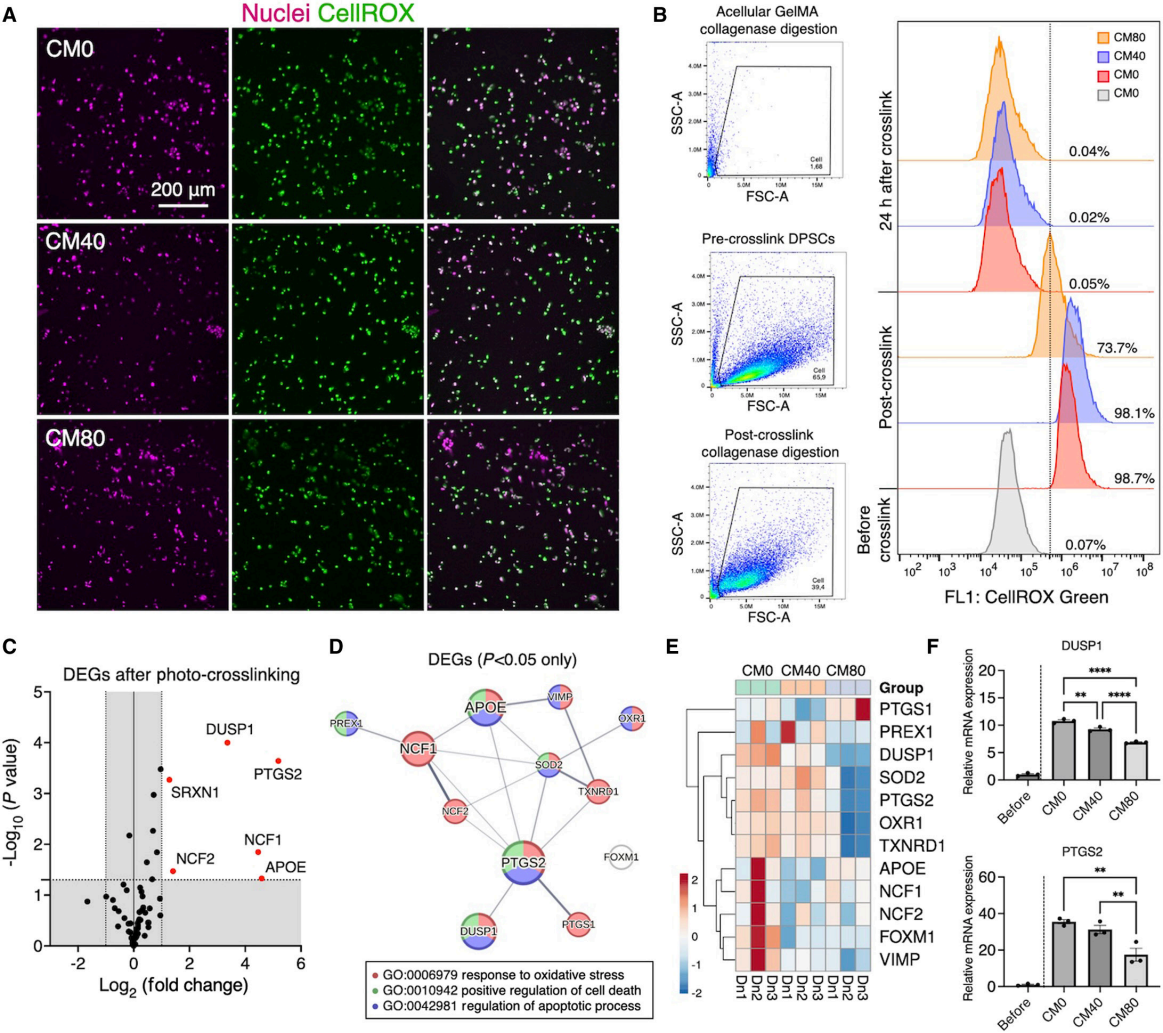
Cellular oxidation during photocrosslinking of GelMA hydrogel rescued by DPSC-CM. (A) Visualization of oxidated cells (in green) in the hydrogel during photocrosslinking. (B) Quantification of oxidated cells by flow cytometry. In the presence of the photoinitiator, over 98% of the cells were oxidated (i.e., labeled with CellROX) during photocrosslinking, which were rescued by DPSC-CM at the high concentration (80%—CM80) by 25%. (C to E) Gene expression array of genes involved in oxidative stress responses and differentially expressed genes (DEGs). (F) In particular, DUSP1 and PTGS2 were highly up-regulated by the photocrosslinking process, which were notably suppressed by the presence of DPSC-CM. *P < 0.05, **P < 0.01, ****P < 0.0001 (Tukey’s multiple comparisons test).

Redox homeostasis disrupted by photocrosslinking and antioxidant properties of CM in the hydrogel were further evaluated. Upon photocrosslinking, the majority of DPSCs were found to be oxidated (Fig. 4A). However, functionalizing with DPSC-CM distinctly reduced the number of cells subjected to oxidation in the GelMA hydrogel by approximately 25% when the hydrogels was prepared with 80% CM (i.e., CM80) (Fig. 4B) compared to the control medium (i.e., CM0).

To evaluate the early response of DPSCs subjected to the oxidative stress, gene expression levels of oxidative stress-induced inflammatory markers were specifically evaluated by RT-qPCR arrays. After the photocrosslinking process, the cells significantly up-regulated genes encoding various inflammatory modulators, such as DUSP1, PTGS2, SRXN1, NCF1, NCF2, and APOE, which are predicted to be involved in the regulation of cell death and apoptotic processes (Fig. 4C and D). Notably, the presence of CM eased the up-regulation in a dose-dependent manner (Fig. 4E). Particularly, DPSCs showed a 10.7-fold increase in up-regulation of DUSP1 after photocrosslinking (*P <* 0.0001: before versus after photocrosslinking) (Fig. 4F). However, the level of expression decreased in the presence of DPSC-CM, with only 9.24- and 6.86-fold increases in the CM40 and CM80 groups, respectively (*P <* 0.0001, CM0 versus CM40; *P <* 0.0001, CM0 versus CM80). Similarly, the expression of pro-inflammatory PTGS2 increased 35.5-fold in CM0 after photocrosslinking, but this was reduced to 17.41-fold in CM80 (*P* = 0.0017, CM0 versus CM80).

### Impaired post-crosslinking kinetics rescued by DPSC-CM

Subsequently, the cell alternation by photocrosslinking was evaluated through the assessment of cell kinetics, namely, cell viability, apoptosis, senescence, proliferation, motility, and morphology. First, the effect of photoactivation of LAP on DPSCs was preliminarily evaluated in suspension. The measurement of intracellular pH using FITC-conjugated dextran (FITC-Dex) revealed a slight alkaline bias when LAP was introduced to the cell suspension (Fig. S2A). Upon photoactivation, the intracellular pH rapidly shifted toward acidity, gradually neutralizing over a period of 3 h. Concurrently, after 3 h of LAP photoactivation, the granularity of most cells significantly increased, suggesting the initiation of an apoptosis process. In addition, immediately following the photoactivation of LAP, over 20% of the cells were found dead, assessed by PI. However, the presence of DPSC-CM reduced the percentage of dead cells down to 16.7% and 13.4% in the CM40 and CM80 groups, respectively (Fig. S2B). No change was observed in the cells upon photoexposure without LAP.

**Fig. 5.**
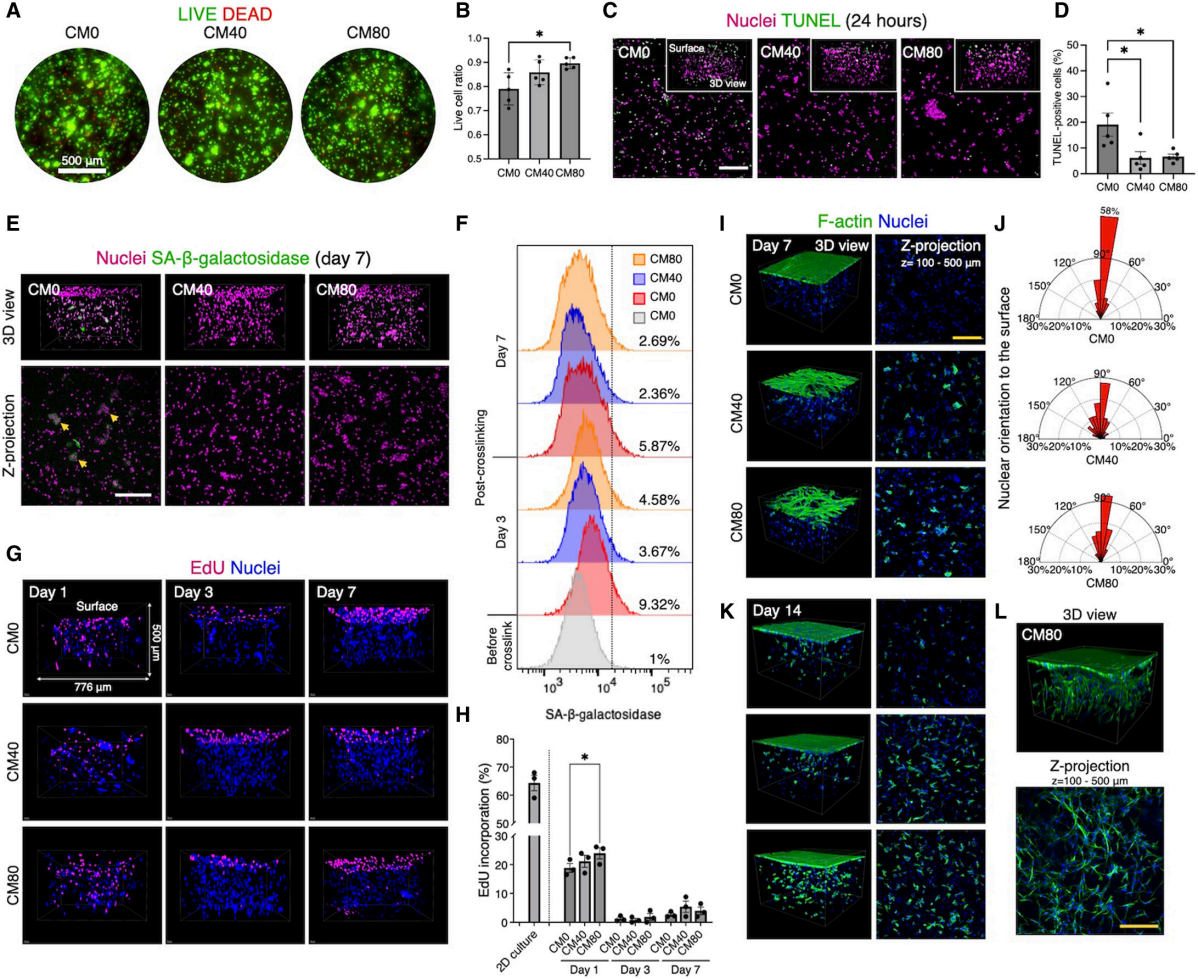
The effects of cellular oxidation by the photocrosslinking process on cell dynamics in the GelMA hydrogel with (i.e., 40%: CM40; 80%: CM80) and without DPSC-CM (i.e., CM0). (A and B) Live/Dead staining and image quantification immediately after photocrosslinking. (C and D) Detection and image quantification of TUNEL-positive apoptotic cells 24 h after photocrosslinking. (E and F) Detection of senescence-associated (SA)-β-galactosidase by microscopy and flow cytometry 3 and 7 d after photocrosslinking. (G and H) Cell proliferation assessment by EdU incorporation and quantification up to 7 d of photocrosslinking. (I to K) Cytochemical staining and quantification to assess 3D cell morphology in the GelMA hydrogel. Cell morphology is displayed as 3D rendered images and z-stack images of z-depth 100 to 500 μm from the surface of the hydrogel. The orientation of nuclei indicates the directional motility toward the hydrogel surfaces when the hydrogel was prepared without DPSC-CM. (L) Representative area with enhanced cell elongation and network formation observed only in the CM80 group sporadically. *P < 0.05 (Tukey’s multiple comparisons test). Scale bars, 200 μm (white) and 100 μm (yellow).

These results were well supported when the cells were encapsulated in the GelMA hydrogel. Following photocrosslinking of the hydrogel, approximately 20% of cells were found dead (Fig. 5A and B). However, DPSC-CM, particularly in the CM80 group, significantly improved cell viability, with approximately 90% of the cells remaining vial (*P* = 0.015, CM0 versus CM80).

TUNEL assay was used to evaluate cell apoptosis 24 h after photocrosslinking provided that apoptosis is a consequence of serial biological cascades [18]. The results revealed that approximately 20% of the cells underwent apoptosis 24 h after photocrosslinking, particularly at the surfaces closed to the light source. Notably, the apoptotic response was clearly prevented by DPSC-CM (*P* = 0.026, CM0 versus CM40; *P* = 0.032, CM0 versus CM80) (Fig. 5C and D). Furthermore, the detection of senescent cells, performed by the detection of senescence-associated (SA)-β-galactosidase, revealed that nearly 10% of the cells became senescent 3 d after photocrosslinking, whereas only 3 to 5% were found senescent in the CM40 and CM80 groups (Fig. 5E and F). The trend was also observed on day 7, where a greater number of cells (>5%) remained senescent in the control medium after photocrosslinking.

Furthermore, cell proliferation in the GelMA hydrogel was evaluated over the period of 7 d by an EdU incorporation assay. Due to the high initial seeding density and relatively stiff hydrogels that favor osteogenesis, cell growth inside the hydrogel was universally suppressed. However, on day 1, the cells were found to be more proliferative in the presence of DPSC-CM in the hydrogel in a dose-dependent manner (*P* = 0.041, CM0 versus CM80) (Fig. 5G and H).

Cell morphology was assessed by observing the formation of a cellular network in the GelMA hydrogel on days 7 and 14 after photocrosslinking, representing the long-term impact of the initial oxidative stress-related cellular impairment. On day 7, cells encapsulated in the GelMA hydrogel without DPSC-CM did not exhibit noticeable filamentous actin nor cell elongation (Fig. 5I). Conversely, cells in the CM40 and CM80 groups displayed discernible, albeit limited, elongated morphology. Noteworthily, cell density on the surfaces of the hydrogel was negatively correlated with the concentration of DPSC-CM. This observation was validated by the quantification of cell axis, where the long axis of nuclei was mostly perpendicularly oriented to the surface of the hydrogel in the CM0 group, indicating the presence of chemotaxis toward the hydrogel surfaces, possibly to avoid the highly oxidated and acidified microenvironment (Fig. 5J). On day 14, most cells in the CM40 and CM80 groups showed clear signs of cell elongation, whereas in the CM0 group only a few cells exhibited limited elongation (Fig. 5K). Furthermore, the DPSCs in the CM80 group demonstrated a spindle morphology building a 3D cellular network sporadically (Fig. 5L).

### Accelerated osteogenic differentiation of DPSC in the functionalized GelMA hydrogel with DPSC-CM

**Fig. 6.**
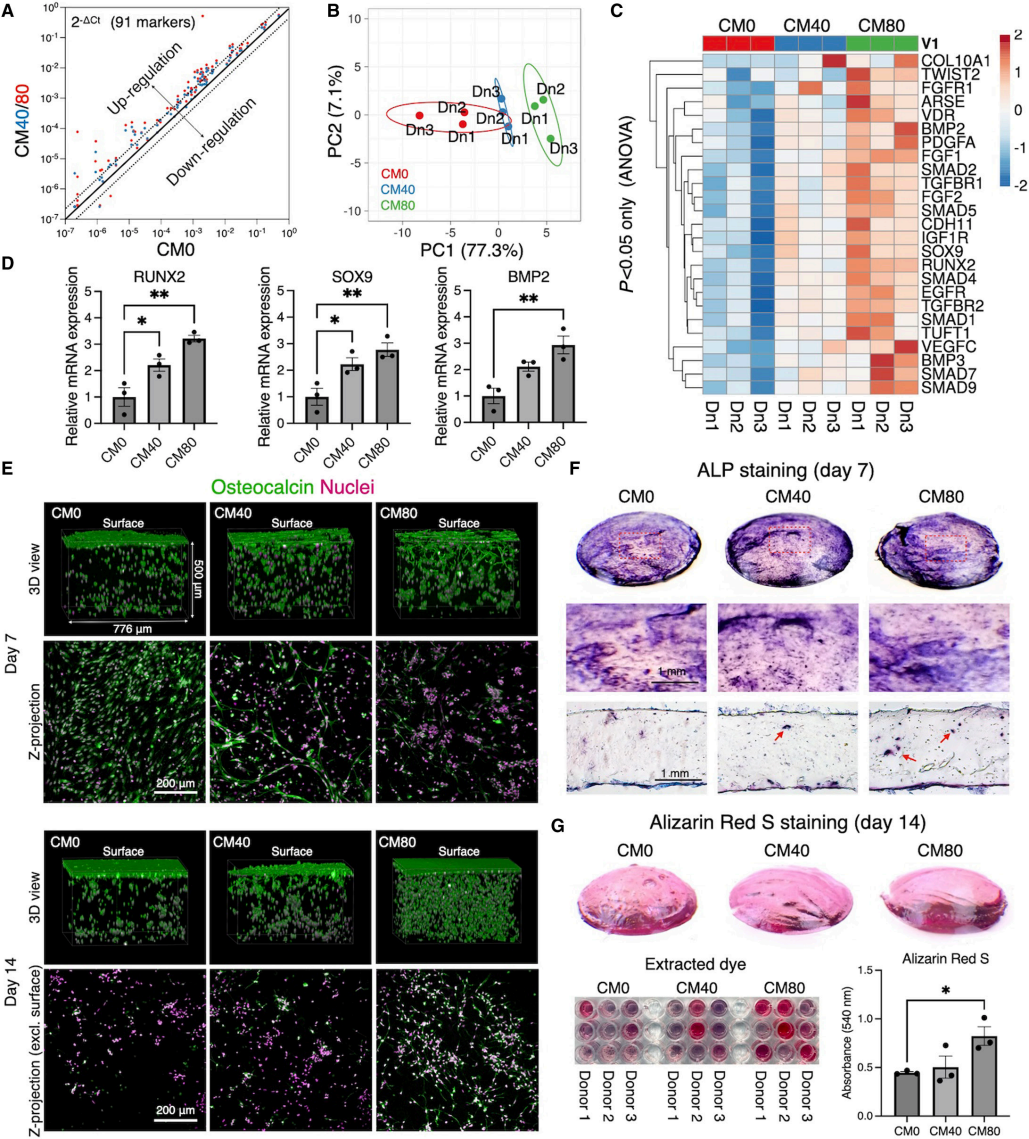
Osteogenic properties of DPSCs in the photocrosslinked GelMA hydrogels with and without DPSC-CM. (A) Scatterplot of 91 osteogenic markers compared to the DPSCs in the control GelMA hydrogel. Expression patterns in the CM40 and CM80 groups were marked in blue and red, respectively. (B) PCA of the osteogenic profile of DPSCs isolated from 3 different donors, showing a population shift in a concentration of the CM-dependent manner. (C) Heatmap of significantly up-regulated osteogenic genes in the presence of DPSC-CM at 40 and 80% concentration. (D) RT-qPCR analysis of key osteogenic and chondrogenic genes, showing that DPSC-CM significantly improved the osteo/chondrogenic properties of the cells in the hydrogels. (E) Immunofluorescent staining of osteocalcin secreted by DPSCs in the GelMA hydrogels with and without DPSC-CM. (F) Macro- and microscopic images of ALP staining, showing the presence of colonies with high ALP activity not only on the hydrogel surfaces but also inside the hydrogels particularly in the CM80 group. (G) Alizarin Red S staining and its quantification, showing that mineralization inside the hydrogel improved CM-dose dependently. *P < 0.05, **P < 0.01 (Tukey’s multiple comparisons test).

To assess the functionality of DPSCs in photocrosslinked GelMA hydrogels with and without DPSC-CM, their osteogenic differentiation was evaluated by culturing the cell-laden constructs in the osteogenic medium up to 14 d. The gene expression array, which targeted 91 genes involved in osteogenesis, revealed significant up-regulation in a dose-dependent manner of DPSC-CM after day 7 of the induction, consistent among DPSCs from 4 independent donors (Fig. 6A and B and Fig. S3). Among these 91 genes, 26 osteogenic genes were significantly up-regulated in CM40 and CM80 groups across all tested donors (Fig. 6C). These included Runt-related transcription factor 2 (RUNX2), SRY-box transcription factor 9 (SOX9), TGF-β superfamily members and their receptors and effectors (BMP2, BMP3, SMAD1, SMAD2, SMAD4, SMAD5, SMAD7, SMAD9, TGFBR1, and TGFBR2), and FGF family members and their receptors (FGF1, FGF2, and FGFR1). In particular, the key transcription factors for osteogenic differentiation, RUNX2, and for chondrogenic differentiation, SOX9, were both significantly up-regulated, with expression levels approximately 2- and 3-fold higher in the CM40 and CM80 groups, respectively (Fig. 6D). Additionally, similar up-regulation trend was observed in the chondrogenesis and osteogenesis regulator, BMP2.

The resulting osteogenic functionality was evaluated by osteocalcin secretion, ALP activity, and biomineralization. The cells in the functionalized hydrogels, particularly in the CM80 group, secreted a significantly greater amount of osteocalcin compared to the CM0 group on days 7, which resulted in enriched network formation of extracellular matrix on day 14 (Fig. 6E). No significant difference in ALP activity was initially observed between the groups after staining in the bulk constructs (Fig. 6F). However, histological sections revealed clusters of cells with strong ALP activity located internally, particularly in the CM80 group, while only the surfaces were stained in the CM0 group. Alizarin Red S staining showed that highly enhanced mineralization was highly enhanced in the CM80 group compared to the CM0 group (*P* = 0.0478, CM0 versus CM80) (Fig. 6G).

### Improved biocompatibility of the cell-laden GelMA hydrogel functionalized by DPSC-CM

**Fig. 7.**
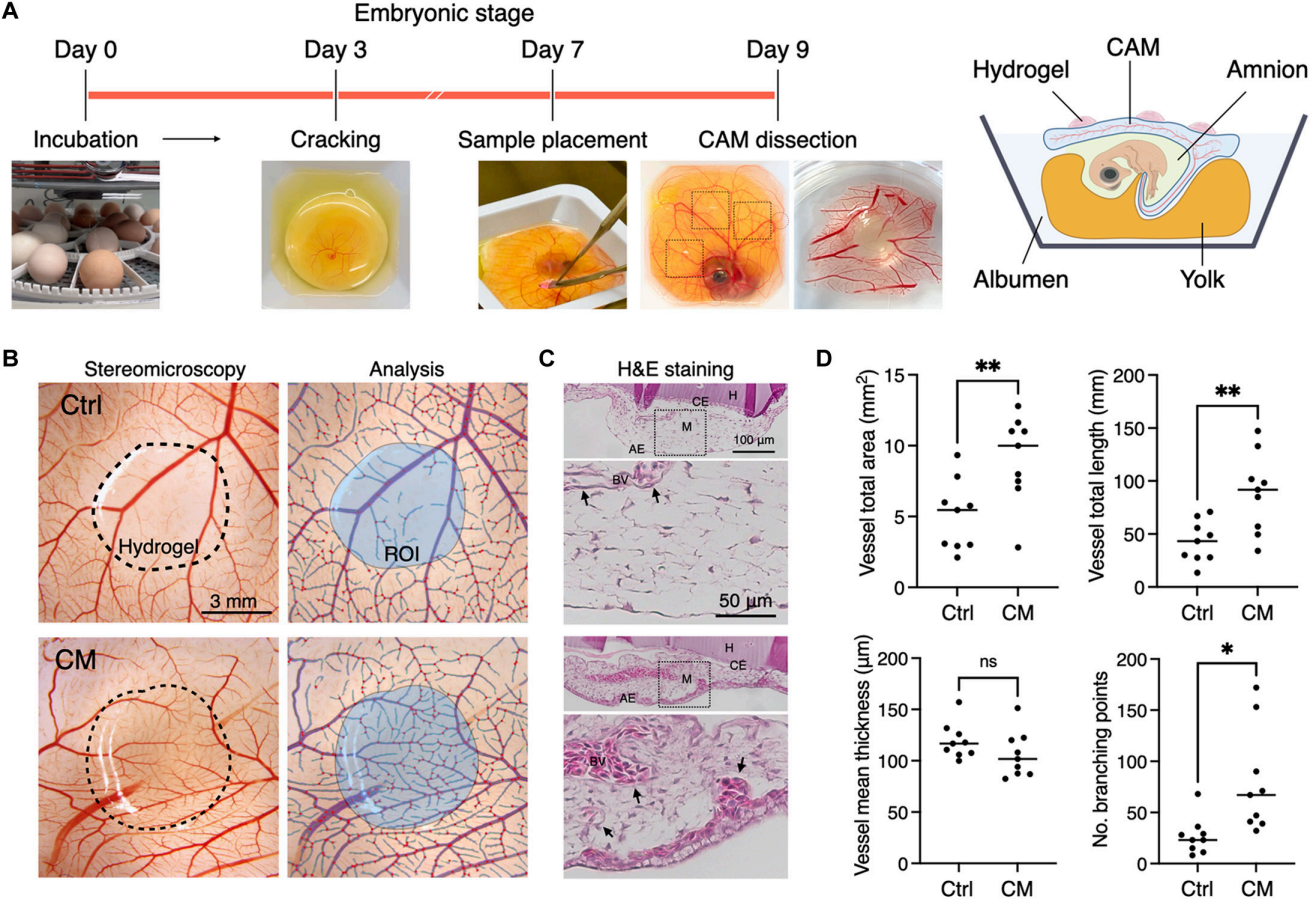
Ex ovo chorioallantoic membrane (CAM) assay for biocompatibility assessment using cell-laden GelMA hydrogels with and without DPSC-CM. (A) Experimental timeline and schematic illustration of the CAM assay. (B) Stereomicroscopic images and region of interest (ROI) for image analysis of vasculature underneath the hydrogels. (C) Hematoxylin and eosin (H&E) staining of CAM, showing atrophied tissue structures when freshly prepared cell-laden GelMA hydrogels in the control medium were cultured on the CAM. The presence of CM rescue the formation of normal tissue formation and vasculogenesis. (D) Quantification of vasculature. Arrows indicate blood vessels. CE, chorionic ectoderm; AE, allantoic endoderm; M, mesenchyme; BV, blood vessels; H, hydrogel. *P < 0.05, **P < 0.01 (Student’s *t* test).

The CAM assay stands as a valuable implementation of the 3R (Replacement, Reduction and Refinement) principle in animal experimentation, offering a rigorous means to evaluate the biocompatibility and angiogenic potential of biofabricated constructs. As a model of implantation, cell-laden GelMA hydrogels were placed on CAM immediately after photocrosslinking and dissected after 48 h for further analyses (Fig. 7A). After placing the samples on the CAM, all embryos remained vital up to CAM dissection. However, microscopic observation indicated that the cell-laden GelMA hydrogels with control medium caused local ischemia selectively underneath the CAM (Fig. 7B). In contrast, the hydrogel prepared with DPSC-CM (i.e., CM80) notably preserved microcapillary and vasculogenesis. Histological observation demonstrated the improved formation of microvessels as well as large vessels under the samples with DPSC-CM compared to the control samples (Fig. 7C). Notably, the stroma underneath the control samples clearly showed sclerotic necrosis-like degeneration, indicating the deterioration of biocompatibility of the GelMA hydrogel. The preservation of microcapillary was confirmed by quantification of vessels total area (*P* = 0.0075), length (*P* = 0.0049), and the number of branching points (*P* = 0.010), showing the presence of denser and finer microcapillaries in the samples with DPSC-CM (Fig. 7D).

## Discussion

The secretome produced by MSCs is gaining significant attention as a promising therapeutic agent in regenerative medicine and dentistry due to its numerous beneficial properties. It includes growth factors, cytokines, chemokines, extracellular matrix proteins, extracellular vesicles, and microRNAs, which have been shown to exhibit anti-inflammatory, pro-mitogenic, immunomodulatory, neuroprotective, and antioxidative effects [6,19]. For example, DPSC-CM has been most frequently tested for its regenerative effects on neurological disorders due to the neural crest origin of DPSCs, followed by its applications in promoting angiogenesis and revascularization, and the regeneration of the dentin–pulp complex, periodontal tissue, and bone. In addition, its therapeutic potential has been suggested for diabetic disorders, cardiovascular diseases, lung diseases, and alopecia [20,21]. Compared to CM produced by BMSCs and adipose-derived MSCs (ASCs), DPSC-CM reportedly exhibit greater potency in terms of neurogenesis [22], neuroprotection [23], angiogenesis [24], osteogenesis [25], and cell migration [26]. Furthermore, studies have reported that DPSC-CM exhibits greater anti-apoptotic potential, highlighting the therapeutic potential of DPSC-CM in regenerative therapy [20,21]. Among CM from dental stem cells evaluated for neural regeneration potential, DPSC-CM demonstrates superiority, akin to CM from stem cells from apical papillae (SCAP-CM) and that derived from human deciduous exfoliated teeth (SHED-CM), surpassing that of periodontal ligament stem cell-derived CM (PDLSC-CM) [27,28]. Comparing to forementioned targets, the antioxidative effects of CM, particularly produced by dental stem cells, seem to be underdocumented. In the present study, while the nature of most proteins can be antioxidative due to their antioxidative amino acid composition (i.e., cysteine, methionine, tyrosine, and tryptophan) and their 3D structure facilitating electrons or hydrogen atom exchange, 91 proteins in DPSC-CM were identified as antioxidants, with enzymatic antioxidants such as PRDX family being particularly enriched. Compared with BMSC-CM, our preliminary MS-based proteomics analysis revealed that the enrichment of these 91 antioxidants was greater in DPSC-CM (Fig. S4). Furthermore, a donor-specific analysis showed that antioxidant proteins, including PRDX 1–6 and SOD1, were significantly more abundant in DPSC-CM, followed by PDLSC-CM, than in BMSC-CM, despite noticeable variations among donors (Fig. S5). These findings suggest that the higher metabolism of dental MSCs, being constantly exposed to mechanical and thermal stress, compared to BMSCs may account for the increased abundance of antioxidant proteins in DPSC-CM. However, a systematic comparison of protein profiles in CM from various sources is still lacking. Ideally, a donor-matched comparison is essential for identifying the optimal source for targeted repair and regeneration.

To regenerate a tissue through tissue engineering approaches, cells are often placed within hydrogels, which act as both a cell carrier and a scaffold. Among the various hydrogels available, GelMA is a popular choice due to its biocompatibility, tunability, and versatility. Proposed biomedical applications of GelMA hydrogel include, but are not limited to, tissue engineering of bone, cartilage, skin, vascular structures, tendons, and muscles, as well as neurofibers. These applications can take the form of 3D hydrogels, injectable hydrogels, 3D-printed hydrogels, microspheres, and fibrous scaffolds [1,29]. A significant advantage of its photocrosslinkable property is that it enables rapid and simple crosslinking in the presence of photoinitiators and allows precise control of both the spatial and temporal aspects of the crosslinking reaction [1]. However, although neither GelMA nor LAP itself is considered cytotoxic, the photoinduced crosslinking is associated with toxicity due to generated free radicals, resulting in oxidative damage to cellular components [2,30]. In particular, mitochondria are intrinsically susceptible to oxidative stress, which promotes mitochondrial fusion and fission during homeostasis [31]. These processes maintain mitochondrial function and integrity by facilitating the exchange of mitochondrial contents, such as proteins, lipids, and mtDNA while segregating damaged portions of the mitochondria for removal through mitophagy. However, excessive oxidative stress has the potential to disrupt the delicate balance between ROS production and the antioxidant defense mechanisms designed to neutralize these reactive molecules and maintain homeostasis. This imbalance is particularly pronounced when cells are subjected to a robust influx of free radicals, causing oxidative damage to proteins, lipids, and DNA [12]. Concurrently, free radicals act as signaling molecules, triggering multiple signaling cascades through redox-sensitive NF-κB, activator protein-1 (AP-1), Janus protein kinase (JAK), p38 MAPK, and p53, determining cell survival and fate [12]. The RNAseq result in the present study clearly showed robust cellular responses to oxidative stress after crosslinking of the hydrogels, particularly represented by dynamic mitochondrial and ribosomal modulation. Interestingly, the presence of DPSC-CM notably altered the gene expression pattern, and while a total number of both up-regulated and down-regulated genes increased in the samples with DPSC-CM, most genes up-regulated in the control group were, in fact, down-regulated in the presence of DPSC-CM. This observation clearly indicates that DPSC-CM modulated stress responses, actively alleviating oxidative damage and enhancing damage repair ability.

The present study demonstrated that, following crosslinking, approximately 20% of the cells died immediately, another 20% underwent apoptosis after 24 h, and 10% entered senescence after 7 d due to oxidative damage. This led to a notable loss of viable and functional cells over time. Concurrently, we observed the increased expression of factors that play a central role in redox homeostasis such as DUSP1 and PTGS2, following photocrosslinking. However, loading DPSC-CM in the hydrogel, particularly at an 80% concentration, was able to mitigate oxidative stress, thus preventing cells from undergoing apoptosis and senescence by suppressing the degree of oxidation and stress response. The supportive effect of loading DPSC-CM was observed not only during the early time phase, i.e., within 24 h, but also after day 7, resulting in greater cellular functions represented as cell proliferation, elongation, and differentiation. Although the study primarily focused on testing the osteogenic differentiation of DPSCs, an enhanced differentiation capacity to other lineages is also anticipated due to the overall improvement in cell health. These results agree on a previous observational study in an in vitro ischemic model, which demonstrated that DPSC-CM exhibited a superior ability to suppress cellular damage caused by hydrogen peroxide and resulting proinflammatory cytokine expression compared to BMSC-CM [32]. It is unclear whether this improvement can be attributed to the direct effect of pro-growth and pro-differentiation factors present in CM or can be explained by the fact that fewer cells were affected during photocrosslinking. However, given that the liquid component in the hydrogel is gradually diffused and exchanged during culture, it is reasonable to assume that the enhanced functionality was due to the improved initial defense mechanisms against ROS and damage repair by CM. This suggests that incorporating DPSC-CM into photocrosslinkable hydrogels may not only reduce the initial number of encapsulated cells required for effective regeneration but also enhance the regenerative potential of cell-laden constructs.

With these biological modulations induced by the photocrosslinking process, we subsequently assessed the biocompatibility of cell-laden GelMA hydrogels at the tissue level. We hypothesized that the hydrogels would still be accepted by the recipient tissue after implantation as reported previously in in vivo rodent models [33,34]. To test this, a CAM assay was employed as a model of implantation known for high sensitivity and real-time visible observation capabilities superior to the traditional rodent subcutaneous implantation model while adhering to the 3R principle [35]. It, however, unexpectedly demonstrated that freshly photocrosslinked cell-laden GelMA hydrogels might not be biocompatible, as evidenced by the depletion of microvessels (i.e., reduced vessel total area/length and reduced number of branching points) and atrophicus changes beneath the samples within 2 d of implantation. The causes are likely multifactorial, including the acidification of hydrogels following photocrosslinking, the activation of apoptotic signaling pathways, necrosis of encapsulated cells induced by ROS, and delayed crosslinking reactions from unreacted residual LAP. Additionally, the cellular redox reactions involved in ROS detoxification contribute to an acidic environment by producing acidic byproducts. For instance, superoxide radicals are converted into hydrogen peroxide by SOD, which is then further converted into oxygen and water by CAT and GPX [36]. Moreover, increased lysosomal activity to remove damaged organelles and a metabolic switch to anaerobic glycolysis under oxidative stress, leading to increased lactic acid production, further lower the intracellular pH [37]. This underscores the necessity of carefully considering the timing of implantation after a photocrosslinking process. Notably, the cell-laden hydrogel made of DPSC-CM mitigated the adverse effects, allowing for normal vasculature development beneath the samples at the given CM concentration. While the result showed that CM prevented the inhibition of vasculogenesis rather than promoting it, further enhancement of angiogenesis and vasculogenesis may be expected by increasing the dose and concentration by, but not limited to, centrifugation and ultrafiltration [38,39]. It is to be noted that the present study focused on the mitigation of adverse effect of the common biofabrication method on biocompatibility and did not address primarily into the potential long-term clinical significance of cell-laden GelMA hydrogels with and without DPSC-CM. Beyond biocompatibility and the modulation of the encapsulated MSCs presented in the study, further preclinical studies are needed to evaluate host responses, including safety, regenerative potential, inflammatory responses, and immune responses, to thoroughly understand the therapeutic implications.

Although the photoactivation of LAP is limited to periods of light exposure, its effects can be enduring. When using a photocrosslinkable system in clinical applications, it is crucial to consider its long-term safety such as mutagenicity and tumorigenicity. While LAP itself is generally regarded as biocompatible, its photoexcited form may pose potential safety concerns. For instance, the guanine base in DNA is highly vulnerable to oxidative stress, resulting in the formation of 8-oxoguanine. This compound is known to induce G to T transversions, a type of point mutation often linked with cancer [40,41]. Furthermore, increased ROS production is known to activate pro-tumorigenic signaling, enhances tumor cell survival and proliferation, and drives DNA damage and genetic instability [42]. Although no studies have yet reported the mutagenicity or tumorigenicity caused by light-activated LAP, long-term in vivo evidence remains limited. While DPSC-CM clearly mitigated the response to ROS, it was perhaps not sufficient to conclude that it did eliminate the presence of ROS. To divert the potential risks associated with free-radical-based crosslinking, a novel strategy known as radical-free (RF) photocrosslinking has recently emerged for tissue engineering applications [43]. This approach employs an RF photo-uncaging mechanism and a base-catalyzed click reaction between thiols and electron-deficient alkene groups. As a result, RF photoresin gelation occurs without generating reactive ROS [43]. This novel method still lacks extensive biological evaluation and has not been widely tested on various materials including GelMA, leaving its full applicability largely unknown. However, the RF approach has the potential to shift paradigms in light-mediated biofabrication, even as efforts will continue to mitigate oxidative stress in free-radical-based methods.

Anticipation for the efficacious utilization of MSC-CM is grounded in its potential therapeutic effects including anti-inflammatory, pro-angiogenic, and antioxidant properties and the acceleration of tissue repair and regeneration. Previously relegated as a laboratory waste with little attention, CM has emerged with recognized potential for clinical applications [19]. As of 2024, the clinical landscape has seen a surge in interest, with over 100 registered clinical trials using CM for therapeutic purposes listed on ClinicalTrials.gov (National Library of Medicine, USA). This reflects the growing interest in translating the therapeutic potential of CM into clinical applications. Nevertheless, before advancing a stem cell secretome product to the market, numerous considerations necessitate careful scrutiny and resolution. As of 2023, the European Medicines Agency (EMA) considers that CM from expanded MSCs does not fall within the definition of an Advanced Therapy Medicinal Product (ATMP) on the basis that it does not contain or consist of recombinant nucleic acids, nor does it contain cells or tissues [44]. Regarding U.S. regulations, CM is classified as either a biological product under Section 351 of the Public Health Service Act or as human cells, tissues, or cellular and tissue-based products (HCT/Ps) under 21 CFR Part 1271. These classifications dictate adherence to distinct regulatory pathways for clinical translation. For instance, under the former classification, oversight is overseen by the Food and Drug Administration’s (FDA) Center for Biologics Evaluation and Research (CBER), necessitating approval through a Biologics License Application (BLA) for a stem cell secretome product. Under the latter, an Investigational New Drug (IND) application is required, unless an exemption applies. Currently, there is no clear regulatory consensus available, neither in the United States nor in Europe, underscoring the necessity for close communication among academics, industry, clinicians, and regulatory authorities. The other necessary consideration is to scale up and standardize the production of MSC-derived CM. For example, spinner flask bioreactors and hollow-fiber bioreactor systems have been employed to either partly or fully automate the production process, aiming to minimize manual handling for standardization and enhance efficiency [45]. The approaches are expected to facilitate mass production while reducing production costs. In contrast, significant work is needed to standardize the production of MSC-CM. Various protocols have been proposed, differing in whether they include concentration and/or filtration steps, the addition of protease inhibitors, the presence or absence of serum, storage conditions, donor tooth, and isolation protocols, and the types of culture media and cell culture protocols used [20]. Additionally, the cell passage number and confluency significantly impact the quantity and quality of CM [46]. Moreover, the optimal concentration for therapeutic effects remains undetermined. A recent systematic review concluded that no clear distinction could be discerned among the available reports regarding the protein concentration or the effects of DPSC-CM, despite a conditioning duration of 48 h being most commonly employed [20]. In our study, we utilized a 48-h conditioning period without concentration, resulting in a protein concentration of approximately 0.2 mg/ml. This approach effectively mitigated oxidative stress on the encapsulated cells. While other studies have reported positive effects on tissue regeneration at concentrations ranging from 1× to 100×, the variability in production protocols complicates cross-study comparisons, making it challenging to identify the optimal protocol [20]. Therefore, the disparity in production protocols needs to be standardized and optimized for clinical translation, although the ideal protocol may vary depending on the target application. Addressing these issues is imperative to pave the way for future off-the-shelf products, providing a standardized and cost-effective approach to harnessing the therapeutic potential of MSC-CM in clinical settings.

Last, the incorporation of CM into hydrogels can profoundly influence their properties and performance in biomedical applications. Higher concentrations of CM often lead to increased viscoelasticity, surface wettability, and initial mechanical strength following crosslinking. This enhancement arises from the presence of proteins, growth factors, and other bioactive molecules within CM, which contribute to improved hydration and mechanical integrity of the hydrogel matrix through physical entanglements and chemical interactions of covalently bound proteins [47–49]. On the other hand, the presence of proteases such as collagenase and other enzymatic activities in CM may accelerate hydrogel degradation over time [50]. These enzymes can degrade the crosslinked structure of the hydrogel, leading to loss of mechanical integrity and potentially disrupting the balance between material degradation and tissue regeneration. Therefore, hydrogel formulations, including the concentration of CM, need to be meticulously designed to achieve desired therapeutic effects while also ensuring optimal mechanical characteristics and biodegradability.

In this study, we provide a comprehensive understanding of cell responses to the photocrosslinkable hydrogels, with a specific focus on cell kinetics and osteogenic differentiation. Our findings demonstrate that DPSC-CM exhibits robust antioxidative properties, which may enhance the therapeutic efficiency of cell therapy when combined with photocrosslinkable hydrogels. Given the versatile regenerative potential and compatibility of both DPSC-CM and GelMA hydrogels, DPSC-CM functionalized hydrogels may be applicable in various biomedical applications beyond bone tissue engineering. Currently, GMP-grade and FDA-approved photocrosslinkable hydrogels are readily accessible in the commercial market. Therefore, the clinical translation of this approach largely depends on overcoming challenges related to scalability, production standardization, and regulatory approval for the use of CM. Further studies are needed to optimize the production processes, establish standardized protocols, and thoroughly evaluate the long-term safety and efficacy of DPSC-CM functionalized hydrogels in various clinical settings.

## Data Availability

The raw data supporting the conclusion of this article are made available upon request to the authors, without undue reservation.
