# Supplementary material for: Harnessing the Antioxidative Potential of Dental Pulp Stem Cell-Conditioned Medium in Photopolymerized GelMA Hydrogels
Source: Biomater Res. 2024 Sep 17;28:0084. doi: 10.34133/bmr.0084 (PMC11406670; doi:10.34133/bmr.0084)
Supplement: Supplementary 1 — Figs. S1 to S5 Table S1 [file bmr.0084.f1.zip › Supporting information Figures.docx]

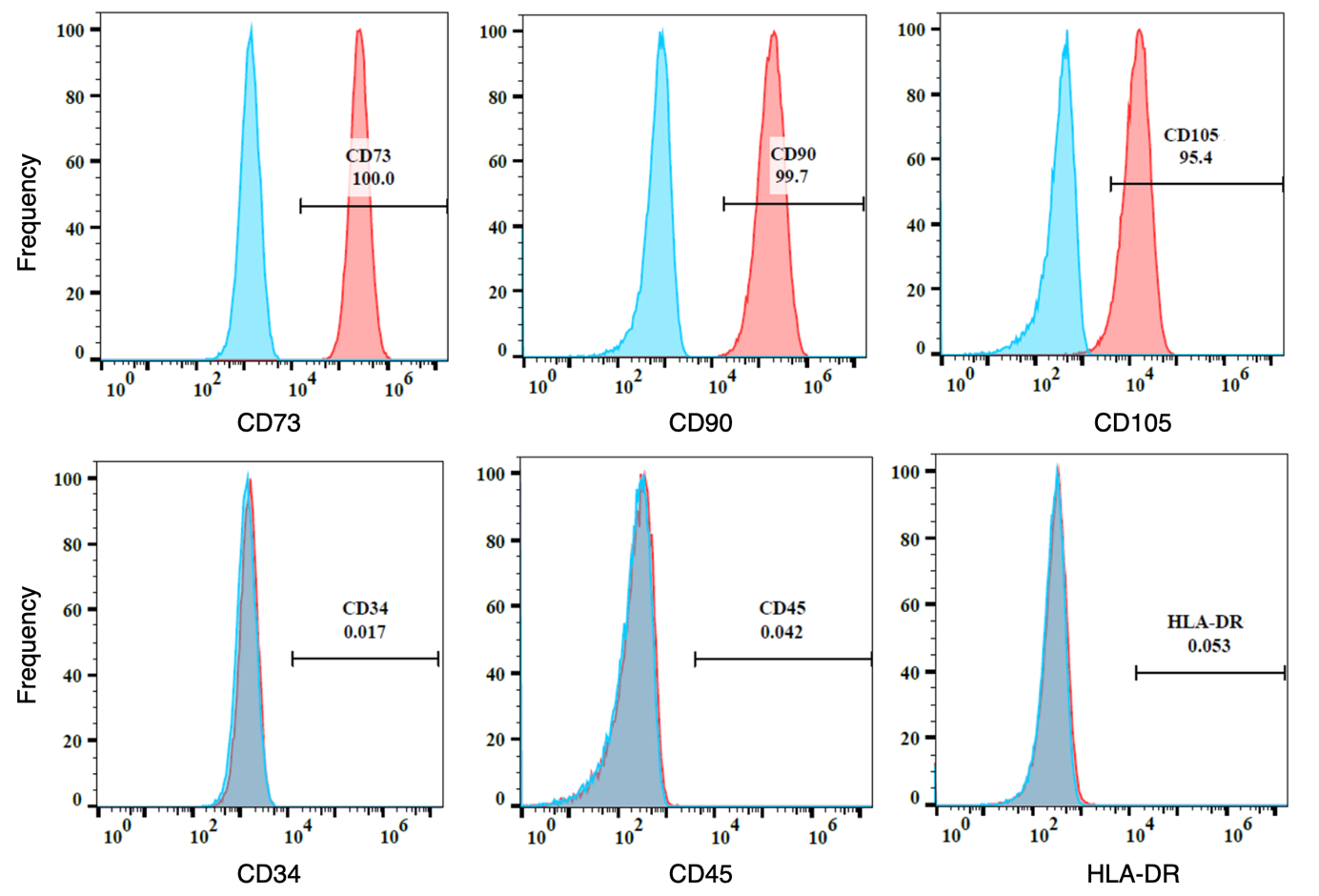


Figure S1 Surface marker characterization of DPSCs by flow cytometry. Blue: isotype controls, Red: samples.


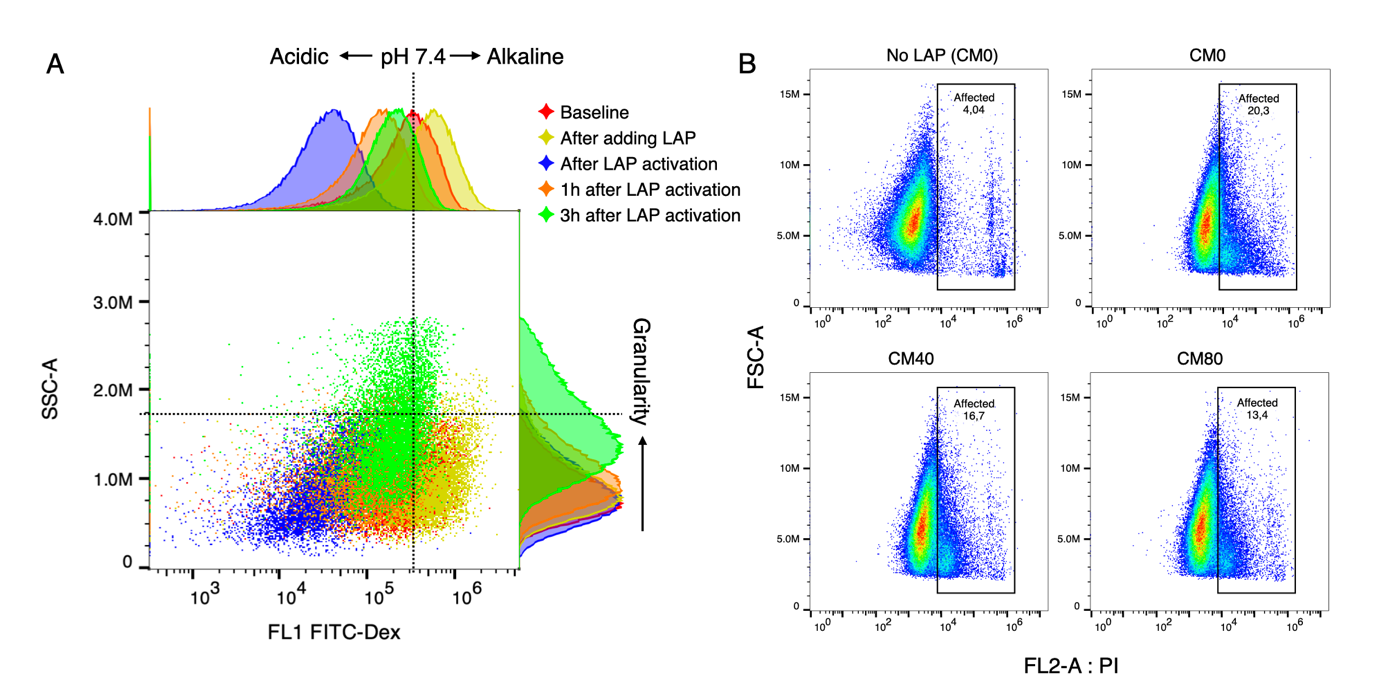


Figure S2 The biological alteration after direct exposure to LAP-inducing irradiation in suspension. (A) Intracellular pH and granularity of DPSCs before and after LAP activation. Intracellular pH, measured by labeling with a pH probe FITC-TRITC-dextran 500 (10 μg/ml; FTD500, TdB Labs AB, Sweden), rapidly dropped after LAP activation followed by gradual recovery to physiological pH over 3 hours. Concurrently, the granularity of the cells increased after 3 hours, suggesting the initiation of apoptosis processes. (B) Flow cytometry analysis of PI-stained necrotic DPSCs after LAP activation with different concentration of conditioned medium.


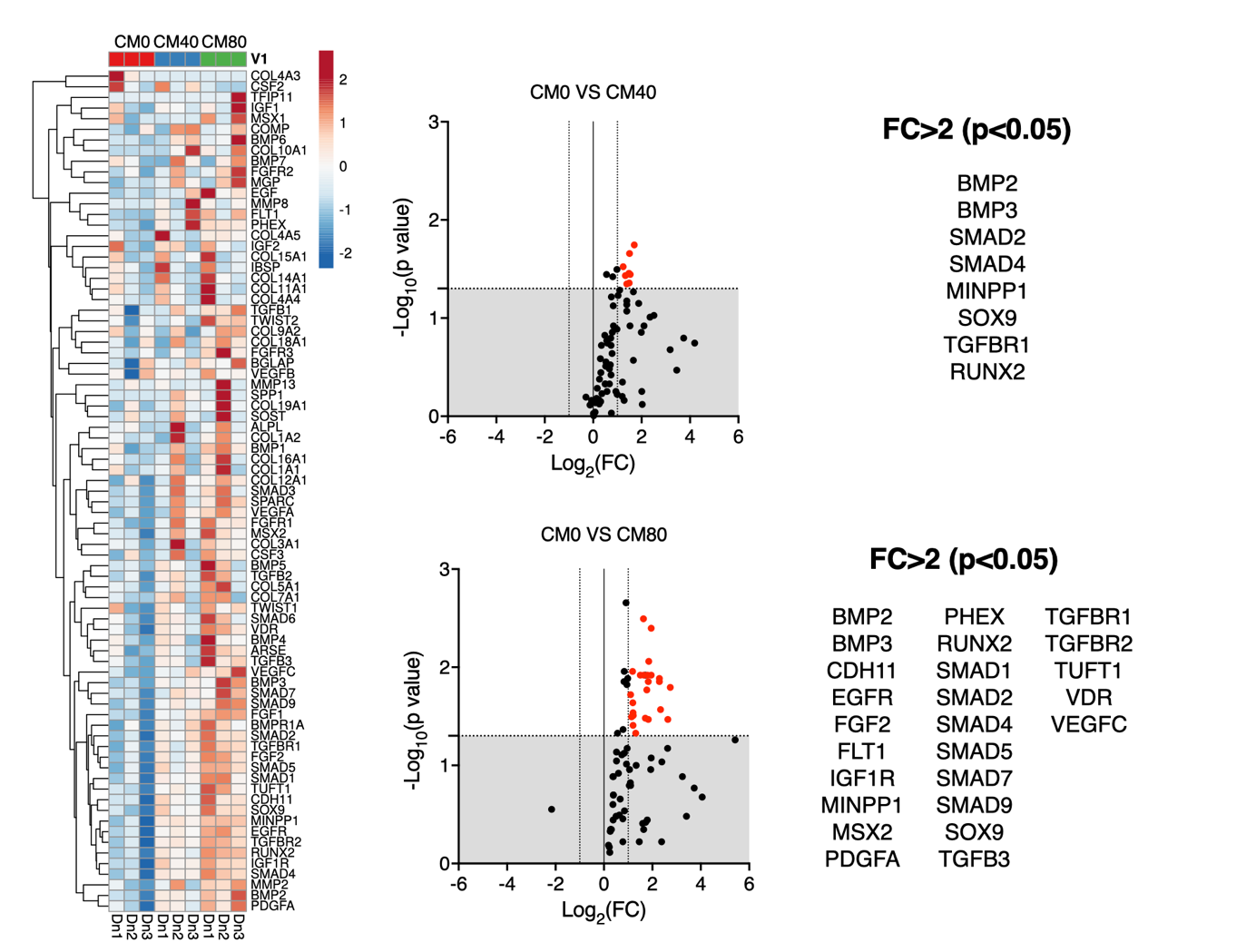


Figure S3 Osteogenic gene expression array of the DPSCs loaded in the GelMA hydrogel with and without the DPSC-conditioned medium (CM). The heatmap chart illustrates the expression of 91 osteogenic markers tested in DPSC-laden GelMA hydrogels functionalized with CM0, CM40, and CM80. Volcano plots compare CM40 and CM80 against CM0, highlighting significantly upregulated genes (fold change > 2) in red and listed alongside.


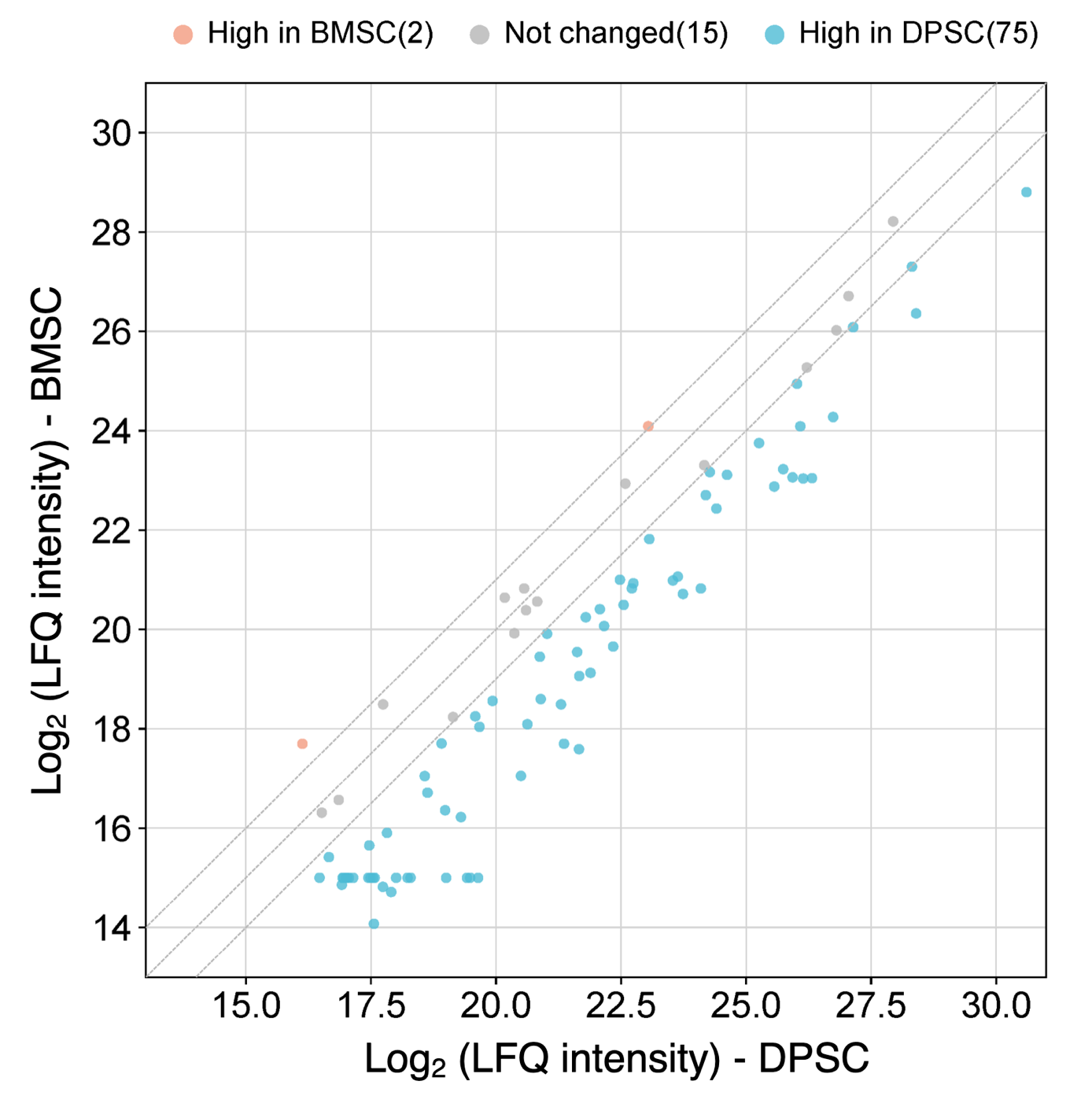


Figure S4 Comparison of the abundancy of 91 antioxidants (see Table S1 for specific protein names) in DPSC-CM and BMSC-CM. Data of DPSC-CM and BMSC-CM are generated from pooled 3 donors and 6 donors, respectively. Among the 91 antioxidants, 75, dotted in Blue, were highly enriched in DPSC-CM compared to BMSC-CM.


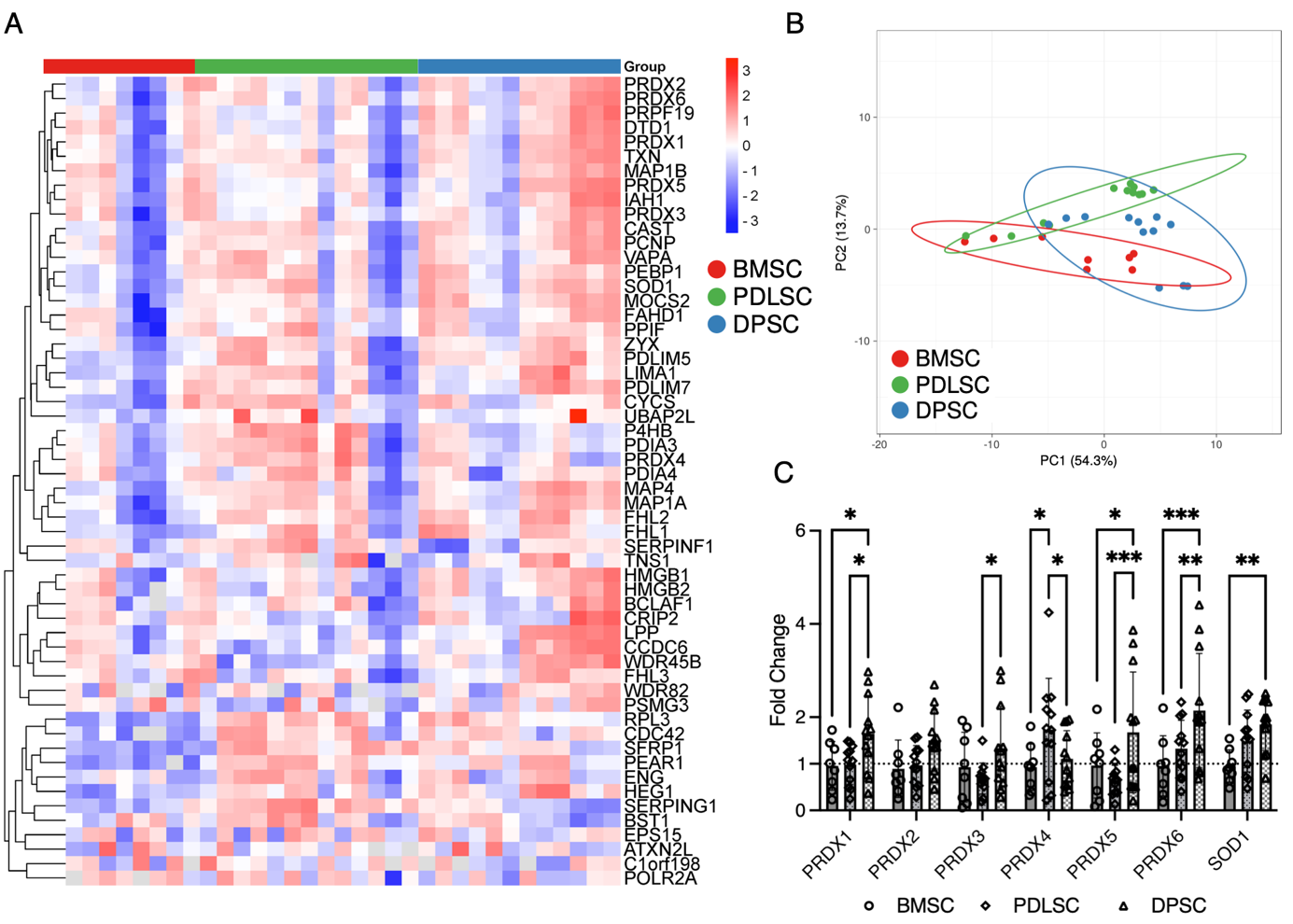


Figure S5 MS-based proteomics analysis of antioxidants in conditioned medium from BMSC, PDLSC, and DPSC. (A) A heatmap image of analyzed antioxidants showing differentially expressed proteins. Each column represents an independent donor (BMSC – 8 donors, PDLSC – 12 donors, DPSC – 12 donors). (B) Principal component analysis plot representing antioxidant protein data from 3 MSC sources. (C) Fold change abundancy of PRDX1-6 and SOD1 in BMSC-CM, PDLSC-CM, and DPSC-CM. Data is presented as mean ± S.D. *p < 0.05, **p < 0.01, ***p < 0.001 (Tukey’s multiple comparisons test)
